# Current Perspectives and Practices of Pet Owners in Türkiye on Animal Care, Nutrition, and Welfare

**DOI:** 10.3390/vetsci12090904

**Published:** 2025-09-18

**Authors:** Salih Çelik, Habip Muruz, Seda Çelik, Mehmet Ferit Can, Mehmet Çelik

**Affiliations:** 1 Tokat Provincial Directorate of Agriculture and Forestry, Tokat 60100, Türkiye; seda.celik@tarimorman.gov.tr (S.Ç.); mcelik@traimorman.gov.tr (M.Ç.); 2 Department of Animal Nutrition and Nutritional Diseases, Faculty of Veterinary Medicine, Ondokuz Mayıs University, Samsun 55139, Türkiye; habip.muruz@omu.edu.tr; 3 Faculty of Veterinary Medicine, Department of Animal Healht Economics and Management, Hatay Mustafa Kemal Üniversity, Serinyol 31000, Türkiye; feritcan@mku.edu.tr

**Keywords:** pet nutrition, pet care practices, pet welfare, feeding practices, pet health and welfare trends

## Abstract

**Simple Summary:**

Pet ownership is rapidly increasing in Türkiye. Most existing research on pet care, nutrition, and welfare comes from developed countries, while information from developing regions like Turkey remains quite limited. This study fills this gap by providing up-to-date, nationally representative data on the daily care and feeding practices of pet owners in Türkiye, as well as their attitudes toward animal welfare. The findings suggest that recent changes in human nutrition—such as a focus on better ingredient quality and foods with additional health benefits—are also reflected in people’s pet food choices. The study also demonstrates the powerful influence veterinarians have on owner decisions and raising awareness of welfare issues. These results are valuable not only for Turkey but also for the global pet food industry, veterinarians, and policymakers working to improve animal welfare through better feeding strategies.

**Abstract:**

Although pet ownership is rapidly increasing in developing countries like Turkey, information on how animals are cared for and fed and on perceived animal welfare remains limited. To address this gap, a survey was conducted with 410 participants from 65 provinces, representing over 80% of the country. The study collected data on pet owner demographics, care and feeding practices, and awareness of animal welfare. The results show that most participants (80.6%) have at least a bachelor’s degree, and most pets (80.9%) were acquired within the last 10 years. Monthly spending on pet care typically ranges from USD 30 to 90. Ingredient quality emerged as the primary factor driving food choices (51%), driven by a growing interest in premium and super-premium products aimed at improving health and well-being. Veterinary clinics play a significant role in shaping feeding decisions. Most pet owners consider their pets family members and feel quite knowledgeable about their welfare and nutrition. The results suggest that recent trends in human nutrition, such as increasing interest in functional foods and higher ingredient standards, are also shaping pet feeding practices, and that closer collaboration between veterinarians and the pet food industry is needed.

## 1. Introduction

Keeping companion animals has been a widespread human practice since the earliest periods of history. Traditionally, both dogs and cats were primarily appreciated for their functional roles: Dogs supported humans in hunting, herding, and guarding, while cats contributed by controlling rodent populations in domestic environments. In contemporary society, however, cats are more often selected as affectionate playmates for children rather than for their hunting skills [1]. When Italian parents were asked to describe their ideal dog, they identified traits such as safe around children, house-trained, healthy, friendly to both people and other animals, obedient, and more [2]. While many reasons (personal preferences, allergy sensitivities, cultural values, etc.) play a role in the choice of breed when it comes to pet ownership, the most important change in pet ownership today, unlike in previous periods, is the way we now see our pets as inseparable members of our families [3]. Pet ownership rates for dogs and cats have been rising steadily worldwide, including across developing economies, with current estimates suggesting that over half of the global population now keeps pets [1]. Currently, domestic dogs (*Canis familiaris*) and cats (*Felis catus*) are the most commonly kept pet species [2]. This increase has been further reinforced by demographic shifts, rising income levels, and social changes associated with the COVID-19 pandemic [1]. Several studies have reported a significant increase in pet ownership during the pandemic period [4,5,6]. The economic size of the global pet industry has reached nearly USD 300 billion and it is expected that the market will grow by an additional USD 50 billion by the end of the next 6 years [7].

According to 2025 records from the PETVET system of the Türkiye Ministry of Agriculture and Forestry, approximately 2.5 million cats and dogs are officially registered in Türkiye [8]. However, this figure likely underrepresents the actual total, as the mandatory microchipping of pets only began in 2021. With the growing popularity of pet ownership, new markets are emerging to meet the expanding demands for pet care, nutrition, and welfare. In recent years, increasing public awareness of health and nutrition has also begun to influence how pet owners approach animal feeding practices and welfare standards. While pet-keeping habits and attitudes towards animal welfare have been examined in numerous international studies, current studies in Türkiye have been addressed to a limited extent, in terms of both their regional nature and the study subjects. This study aims to contribute to the international literature by comprehensively examining the demographic characteristics, cat and dog care and feeding practices, and attitudes towards animal welfare of pet owners in Türkiye along with some economic aspects of the matter.

## 2. Materials and Methods

This research was conducted in Türkiye from June 2024 to January 2025, with approval number 04/43 issued by the Ethics Committee of the Social and Human Sciences at Tokat Gaziosmanpaşa University on 11 June 2024. Data were collected through an online survey prepared using the Google Forms platform. The survey form was disseminated to cat and dog owners via veterinary clinics operating in all 81 provinces of Türkiye and through Animal Health Branch Directorates affiliated with the Ministry of Agriculture and Forestry. Prior to participation, a brief explanation of the study’s purpose was provided to participants, and only individuals aged 18 and over were permitted to participate. Informed consent was obtained from all participants who participated voluntarily, and it was made clear that the data would be used solely for scientific purposes.

The survey form was designed to quantitatively evaluate the daily practices of cat and dog owners regarding animal care, feeding routines, and their perceptions and attitudes towards animal welfare. The questionnaire comprised 28 items, organized under four thematic sections—most questions employed multiple-choice formats and five-point Likert-type response scales. The first part involved collecting information on the socio-demographic characteristics of the participants, including their age, gender, level of education, type of residence, pet ownership status, and monthly household income. The second part covered the basic characteristics of the pets owned, including type, number and method of acquisition. The third section addressed pet owners’ feeding preferences, including food type, feeding frequency, preferred feeding strategies, and purchasing habits. The final section aimed to evaluate participants’ knowledge of animal welfare and their engagement in social and physical activities with their pets. The clarity and face validity of the questionnaire were assessed through a pilot study involving 12 pet owners. During the survey, participants with more than one pet were asked to base their responses on their first pet.

Statistical analyses were conducted using IBM SPSS Statistics, version 25.0, and the Python programming language, version 3.10, utilizing the Pandas and SciPy libraries. Descriptive statistics were calculated to summarize the characteristics of the participants and pet-related variables. Items measuring attitudes on a five-point Likert scale (1 = strongly disagree, 5 = strongly agree) were analyzed by calculating the mean scores and comparing them across demographic subgroups. In addition to descriptive analyses, inferential statistical tests were performed to examine group differences and relationships between variables. The chi-square (χ^2^) test was applied to analyze categorical variables. The Mann–Whitney U test was used to compare two independent groups, while the Kruskal–Wallis H test was employed for comparisons involving more than two independent groups. A two-tailed significance level of (*p* < 0.05) was applied for all statistical tests.

## 3. Results

### 3.1. Demographic Characteristics of Participants and Pet Ownership

**Figure 1:**
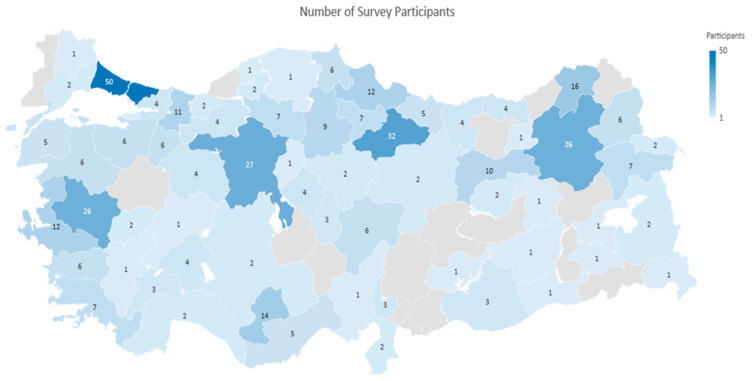
Geographic distribution of survey respondents across Turkish provinces (*n* = 410).

Upon completion of the survey, a total of 410 valid responses were collected. All participants were cat or dog owners and represented 65 of Türkiye’s 81 provinces, corresponding to a national coverage rate of 80.2% (Figure 1). The provinces with the highest number of responses were Istanbul (*n* = 50), Ankara (*n* = 32), and Izmir (*n* = 26). The concentration of responses in these major cities can be explained by the increase in population and pet ownership associated with urbanization. Our survey received a limited number of responses from Kahramanmaraş, Hatay, Adıyaman, and Malatya, which were affected by the 2023 earthquakes in Türkiye. This decline in participation is thought to be related to post-disaster population migration and a possible decrease in pet ownership rates.

Of the participants, 58.8% were female and 41.2% were male. There was no statistically significant difference between the gender ratio of the participants and that in the National Pet Registration System (PETVET) data (χ^2^ = 1.93; *p* = 0.164). This result supports the reliability of the study in terms of gender representation. Examining the age distribution, the most significant proportion of participants (49%) were in the 26–40 age group, followed by the 41–65 age group (37.3%). Examining the education level data shows that 55.6% of participants have a bachelor’s degree and an additional 31.2% have postgraduate qualifications (master’s or doctoral level). These findings demonstrate that pet ownership in Türkiye is more prevalent among women, working-age individuals, and individuals with a higher level of education (Table 1).

The study found that the vast majority of pet owners (78.7%) live in apartments, with 6.8% living alone and 23.2% living in a household with five or more people. Data on household income levels indicate that 5.4% of participants reported an income below the minimum wage (approximately USD 500) as of 2024, which is the valid minimum wage in Türkiye. More than half of the participants (50.2%) stated that their annual expenses for the care and feeding of their pets average between USD 360–1080 (Table 2). In this study, specific categories of care expenses, such as food or veterinary costs, were not distinguished separately; consequently, the reported expenditures represent the total pet care investment.

Of the participants in the study, 82.9% had adopted a pet in the last 10 years, and the majority were cat owners (71.2%). Eleven per cent of the participants stated that they had both a cat and a dog at home. In general, it is seen that the majority of pets are neutered (71.5%). However, this rate varies significantly according to the animal species; while the neutering rate is high at 78.6% in cats, this rate is quite low at 41.6% in dogs. (*p* < 0.001) [OR] = 5.16, 95% CI: 2.77–9.60) (Table 3).

### 3.2. Preferences Regarding Animal Feeding, Care and Related Expenditures

The preferences of pet owners participating in the study regarding the feeding of their cats and dogs are presented in Table 4. The vast majority of pet owners (47.8%) reported feeding their pets commercial food. This rate varies between species, and cats are fed commercial food more often than dogs (57.8% vs. 22.2%). The places where commercial food is purchased are the internet (44.9%), veterinary clinics (24.4%), and pet shops (22.3%), respectively, and dry food (95.1%) is purchased most often. The most important factor affecting the purchase of commercial food by cat and dog owners is the content of the food, at a rate of 51%. The second most important factor is the animal’s liking, at 28.1%. When the feeding preferences of pet owners were examined, it was found that 47.8% fed their pets only commercial pet food, while 29.4% used commercial food in combination with home-prepared diets. The rate of those who preferred only homemade or alternative feeding approaches (raw feeding, biologically appropriate raw food (BARF), or cooked natural diets) was 14.2%. In the evaluation made on a species basis, it was determined that the rate of feeding only commercial food was relatively high among cat owners, at 57.6%. In comparison, this rate remained at 23.6% among dog owners. This difference was found to be statistically significant (*p* < 0.001). [OR] = 2.85, 95% CI: 1.57–5.17.

It was determined that 44.2% of those using commercial food preferred super-premium (commercial diets formulated with high-quality ingredients and enhanced nutrient profiles), 38.3% preferred premium (commercial diets of good quality but lower nutrient enhancement than super-premium), and 14.6% preferred the standard (economic) segment food (basic commercial diets with standard nutrient levels), based on nutritional quality classification. When the factors affecting food selection were evaluated, it was determined that 51.0% of the participants considered the product content, 28.1% considered the animal’s willingness to consume the product, 10.4% considered the veterinarian’s recommendation, 7.2% considered the product price, and 3.3% considered brand awareness. (Table 4).

**Figure 2:**
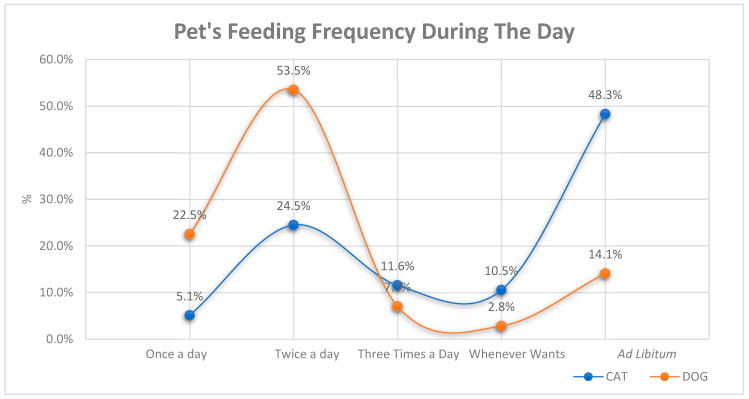
Daily feeding frequency of cats and dogs reported by participants.

In the study, when the daily feeding numbers for cats and dogs were compared, it was found that there were significant differences between the species (*p* < 0.001). [OR] = 4.21, (95% CI: 2.95–6.01). While 48.3% of the participants stated that they fed their cats *ad libitum* (free access), this rate was reported as only 14.1% for dogs. The majority of dog owners (53.5%) reported feeding their animals twice a day, while 22.5% stated that they fed them once a day. (Figure 2). These species-specific disparities in feeding practices are indicative of behavioral and management considerations exhibited by pet owners, thereby underscoring a predilection for structured feeding in canines and a greater degree of flexibility in felines.

### 3.3. Animal Welfare Assessment

**Figure 3:**
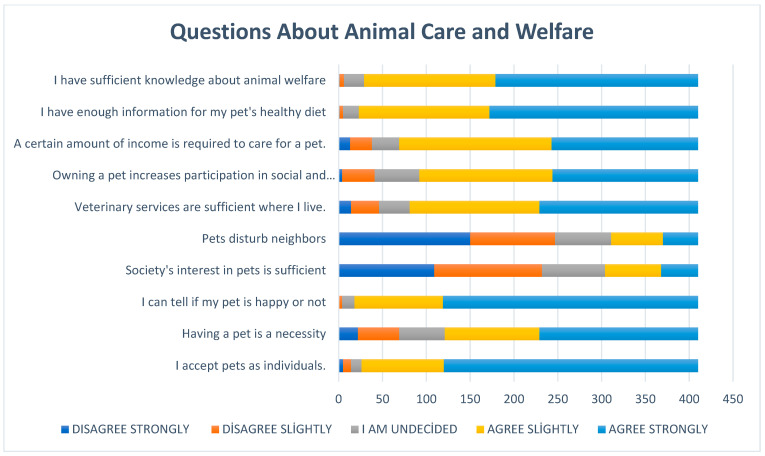
Pet owners’ approaches to animal care, nutrition, and welfare awareness.

A total of 93.6% of the participants defined their pets as “family members”. The high rate of agreement given to the statements “I accept my animal as an individual” and “I can tell if it is happy or not” also supports this approach. The vast majority of participants (94.3%) stated that they have knowledge about nutrition, and 92.9% stated that they have knowledge about animal welfare. On the other hand, it is thought that the interest and sensitivity towards pets in society is insufficient (56.5%). Evaluations regarding veterinary services are more positive. Eighty per cent of the participants stated that they found the veterinary health services in their place of residence to be sufficient (Figure 3).

**Figure 4:**
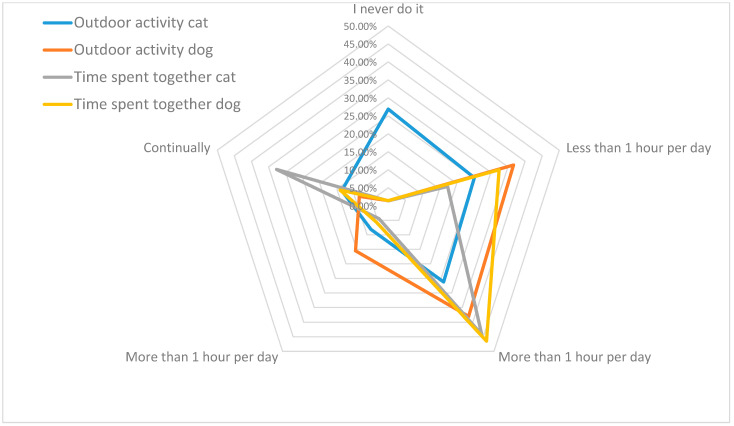
Average daily time spent indoors and outdoors with pets by species.

Two separate questions were asked to assess the daily time participants spent outdoors or indoors with their pets. Data from 45 participants (10.9%) who owned two different species (cats and dogs) at the same time were excluded from the analysis because the species-based distinction in their responses could not be made. Comparisons based on species were made using data from individuals who reported owning only one type of pet (cats or dogs). While 26.8% of cat owners stated that they never take their pets outdoors, this rate was only 1.4% for dog owners. Statistically significant differences were observed in pet owners’ habits of spending time outdoors with their pets, according to species (*p* < 0.05) (Figure 4).

## 4. Discussion

### 4.1. Demographic Data and Pet Ownership

According to figures from the Ministry of Agriculture and Forestry’s Pet Registration System (PETVET) for January 2025, there is at least one cat or dog in 2,483,404 households across Türkiye [8]. In the United States, the percentage of households with dogs is 45.5%, while the percentage of households with cats is 32.1% [9]. In European Union countries, the average pet ownership rate (cats or dogs) is 26% [10]. These data show that the pet ownership rate in Türkiye is significantly lower than in European countries and countries with high ownership rates, such as the United States. However, the process of identifying pets and registering them in official databases began in Türkiye in 2021, and it is estimated that the current PETVET data may not fully reflect the actual ownership rates, as the registration system is still in its initial stages.

Pet ownership is a multifaceted phenomenon that is preferred not only for individual reasons but also for social, economic, and psychosocial reasons. In recent years, a significant increase has been observed in pet ownership rates worldwide [1]. Demographic transformations, increasing living standards, changes in household structure, and especially the social effects of the COVID-19 pandemic, which began in 2019, have played a significant role in this increase [4,5,6,11,12]. The increasing trend in pet ownership around the world in recent years is also parallel to the findings obtained within the scope of our study. While 82.9% of the participants stated that they had adopted a pet (cat or dog) at least once in the last 10 years, it was determined that 42% of this group adopted it in the last three years. These data indicate a significant increase in pet ownership during the post-pandemic period, and the COVID-19 pandemic has also had a transformative impact on pet ownership behaviors in Türkiye.

When the household structures of pet owners are examined, the rate of individuals living alone was found to be 4.1% for dog owners and 7.4% for cat owners. These rates are relatively low compared to those in European countries. For example, in a similar study conducted in Ireland, the rates of dog owners living alone and cat owners living alone were reported as 50.1% and 59.1%, respectively [13]. This difference can be explained by the fact that the household structure in Türkiye is based on a more extended family model. In addition, the rate of those who stated that children were influential in the decision to adopt a pet was 21.4% in our study (Table 2). This situation is evaluated as related to the joint decision-making process within the family rather than an individual decision.

According to the findings of our study, slightly more than half of the participants (50.2%) reported spending an average of TRY 12,000–36,000 (approximately USD 360–1080) per year on the care and feeding of their pets. When compared to data from developed countries, it is seen that annual spending levels for pets are lower in Türkiye. For example, a national study conducted in the USA reported that the annual care and feeding expenses of cat owners were USD 970, and those of dog owners were USD 2021 [14]. It has been reported that these spending levels will reach an average of USD 1516 in the USA by 2024 [8]. Again, annual care expenses are higher in European countries compared to Türkiye (e.g., over EUR 1000 in Germany and the Netherlands). In similar studies conducted by Zhang et al. [15] and Wolf et al. [16], it was reported that expenditures on pets were directly proportional to the increase in personal income. Therefore, income level is an important issue in animal ownership, which directly affects the care, nutrition, and welfare of animals.

When examining the sterilization rates of domestic animals, it has been found that 78.5% of dogs and 40.8% of cats are sterilized. The low sterilization rate among dogs, in particular, reveals that the attitudes of domestic animal owners in Türkiye towards birth control differ from trends in developed countries. While the primary reasons for sterilization—preventing unwanted reproduction, reducing behavioral problems, and lowering the risk of reproductive system diseases—are generally accepted by pet owners, the ethical implications of sterilization and its effects on animal welfare remain a subject of debate at the global level [17]. The relatively low rate of neutering (41.6 per cent) gives rise to significant concerns from both an ethical and a public health perspective, particularly in light of the recent increase in the number of stray dogs in Turkey. This situation can give rise to a number of serious public health problems, including negative interactions with stray dogs (e.g., aggression, traffic accidents) and the transmission of diseases such as rabies, toxoplasmosis, and mange. This situation underscores the significance of awareness campaigns and public policies that promote routine neutering.

### 4.2. Pet Care, Feeding Preferences, and Related Expenditures

Today, pet owners have begun to reflect on the care they provide to their pets, including the content of the foods they consume and their effects on the health and nutrition of their pets [18]. The increasing awareness of pet owners about the health of their animals has led to a rise in diversity in the pet market, making the commercial food purchasing process more complex. Numerous studies on pet nutrition have emphasized that factors such as price, content, and product quality are the most important determinants in food selection [19,20]. Focusing on content and good nutrition, in particular, reveals the direct impact of healthy lifestyle trends on human health and nutrition, as well as their influence on pet nutrition [21]. The findings obtained in our study, consistent with the literature, showed that 51% of the participants stated that they give priority to food content when purchasing pet food. This rate indicates that pet owners in Türkiye prioritize content as the primary criterion when selecting food.

According to the research results, the majority of participants (95.1%) reported giving their pets commercial dry food. This situation reveals that pet owners in Türkiye generally prefer ready-made and commercial products. When dry food usage rates were compared between cat and dog owners, no significant difference was observed (*p* > 0.05). This result indicates that dry food is the primary choice for the nutrition of both cats and dogs. On the other hand, commercial food plays a more significant role in the nutrition of cats. While 97.2% of participants stated that at least half of their cats’ diet consists of commercial food, this rate was 70.1% for dogs (*p* < 0.001). This difference indicates that dog owners prefer home-prepared food more frequently.

The top two places where commercial pet food is purchased are online (44.9%) and veterinary clinics (24.4%). The influence of veterinarians on pet food purchasing preferences is pervasive in Türkiye, similar to studies conducted in other countries [18,21,22,23] The study found that participants based their food preferences on “content quality” (49%, *n* = 201). In contrast, “price” was cited as the primary reason for preference in only 5% of cases. This indicates that price sensitivity is secondary, and that conscious choices are made regarding the health of the animal when selecting food. The quality of commercial pet food is a direct factor influencing the longevity and health of pets [24]. In recent years, there has been an increasing trend among pet owners to pay more attention to the characteristics of the food they purchase for their pets, in order to provide them with a healthy life. This trend has been reported to influence consumers’ purchasing preferences [21]. Similar to recent studies conducted in Italy [18] and Portugal [25], our study found that pet owners prefer super-premium (44.2%) and premium foods (38.3%) containing more functional nutrients for their pets’ health and well-being, compared to economical foods (17.5%). A statistically significant relationship was found between food quality preference and income level (*p* = 0.008). It was observed that individuals in higher income groups tend to prefer premium or super-premium food. However, education level was found to have no significant effect on food quality preference (*p* = 0.180). This indicates that pet food preference is independent of educational level and aligns with income level. The study also examined whether the factors influencing pet food selection differ between cat and dog owners; however, the analysis revealed no significant difference between the two groups (*p* = 1.000).

The digestive systems of cats and dogs differ significantly in some respects. This leads to differences in various factors, including feeding frequency and diet composition [26]. Pet owners are likely to be influenced by a range of factors when determining their pets’ diets, including their preferences, knowledge of their pets’ nutritional needs, pet food ingredients, the effects of marketing strategies, and sources of information related to their pets’ dietary management [27]. There is a statistically significant difference in feeding habits related to meal frequency between cats and dogs, consistent with previous study findings [22,28] (*p* < 0.001). This finding suggests that different feeding practices are adopted based on the physiological and behavioral characteristics of domestic animal species. In particular, the prevalence of ad libitum (free access) feeding in cats (48.3%) is consistent with their tendency to eat small amounts frequently throughout the day. In dogs, feeding is largely structured (53.5% twice a day, 22.5% once a day).

### 4.3. Animal Welfare Approaches

The concept of animal welfare can be summarized as follows: An animal is in good condition if it can comfortably exhibit its natural behaviors in a living environment that is appropriate to its nature [29,30]. In a comprehensive qualitative sociological study conducted by Sanders [31], dog owners began to view their dogs as “unique individuals who are intelligent, empathetic, reciprocal, and aware of the basic rules and roles that govern the relationship” based on their close interactions with them. A total of 93.6% of participants in the study view their pets as individuals. Similarly, in other studies [3,32,33,34,35], the majority of participants identified their animals as family members. According to the results of a survey conducted in the Netherlands in 2021 with 1859 cat owners, more than half of the owners view their cats as part of their family, while more than a quarter view them as children, and a smaller number (14%) view them as pets [36]. Globally, an increasing number of people view their pets as part of their family [35]. This changing perspective toward pets has led pet owners to be more attentive to issues such as their pets’ health, nutrition, and welfare, and to increase their knowledge in these areas.

When pet owners were asked to evaluate society’s attitudes toward pets, 57% of participants stated that society’s interest in pets was insufficient; only 25.8% found the interest sufficient, while 17.2% were undecided. It is thought that news stories frequently appearing in the media in recent years in Türkiye, particularly those involving stray dogs, such as biting incidents, traffic accidents, or social security concerns, may increase the risk of developing negative perceptions toward animals in individuals who have not previously had experience with pets. This phenomenon is also frequently emphasized in the literature [37,38,39]. Considering that animal welfare can only be sustained through social acceptance, institutional support, and individual awareness, it is recommended that accurate information, media guidance, and community-based awareness campaigns be promoted to eliminate such prejudices. Pets act as “social catalysts,” leading to increased social contact between people [34,40] and indirectly enhancing human well-being and health by promoting social interaction with others [34]. Seventy-eight per cent of survey participants reported that owning a pet enhances socialization, and 70.7% stated that owning a pet is truly a necessity. Similarly, increasing social acceptance and evolving cultural attitudes, not only in developed countries but also in Middle Eastern countries such as Saudi Arabia and the UAE, have led to the greater integration of pets into homes, indicating a growing recognition of their role in human well-being and social life [41,42]. In general, people do not adopt pets specifically to improve their health; instead, they value the relationship they have with their pets and the contribution pets make to their overall quality of life [36]. Due to their nature and physical needs, dogs spend more time outdoors than cats. Our study results are consistent with those of a study conducted in Mexico [43]. However, when considering the total time spent together at home and outdoors, it was found that people with cats spend more time with their pets than those with dogs.

The concentration of survey responses in urban areas likely reflects the higher population and pet ownership in these regions. However, pet owners from earthquake-affected and rural areas were underrepresented, which is a limitation of the study. This may have resulted in higher reported levels of education, pet care and feeding expenditures, and animal welfare practices among participants.

## 5. Conclusions

This study examined the attitudes of pet owners in Türkiye towards the care and feeding habits of their animals, as well as their views on animal welfare. The results obtained revealed that ownership rates are related to socio-demographic structure and geographical factors, commercial food is widely preferred in animal feed, and awareness of content and quality is important in food selection. It is recommended that the food sector supports pet owners in making conscious choices by presenting product contents clearly and transparently, and that awareness is increased through informative labels that emphasize the effects of ingredients on health and welfare. Low neutering rates have been identified as an important area requiring intervention. It is recommended that veterinarians share up-to-date information on care and nutrition with pet owners and lead animal welfare activities. Consequently, it is imperative to develop sustainable policies in collaboration across sectors to improve pet nutrition, care, and welfare.

## Figures and Tables

**Table 1:** Demographic characteristics of pet owners participating in the survey (*n* = 410).

Data	Category	Frequency (*n*)	Percentage (%)
Gender of Participants (*n* = 410)	Male	169	41.2
	Female	241	58.8
Pet Owner Age (*n* = 410)	Age 18–25	50	12.2
	Age 26–40	201	49.0
	Age 41–65	153	37.3
	Age 66–79	6	1.5
	Age 80 and over	0	0.0
Pet Owner’s Education Level (*n* = 410)	Primary education	8	2.0
	High school	46	11.2
	Bachelor	228	55.6
	Master’s or Doctorate	128	31.2

The table summarizes the distribution of participants according to gender, age, and education level. Data are shown as frequency (*n*) and percentage (%). Percentages may not total 100 due to rounding.

**Table 2:** Information on the household where the pet owner lives.

Data Answers		Frequency (*n*)	Percentage (%)
Type of House (*n* = 410)	Apartment house	323	78.7
	House with garden	87	21.3
Number of People Living in the House (*n* = 410)	Alone	28	6.8
	2 people	70	17.1
	3 people	101	24.6
	4 people	116	28.3
	5 or above people	95	23.2
Pet Owner’s Monthly Household Income (*n* = 410)	Less than USD 500	22	5.4
	USD 501–1470	140	34.1
	USD 1471–2940	179	43.7
	More than USD 2940	69	16.8
Pet Care and Feeding Expenses at Home (monthly expenditure) (*n* = 410)	Less than USD 30	105	25.6
	USD 31–90	206	50.2
	USD 91–150	65	15.9
	USD 151–300	30	7.3
	More than USD 300	4	1.0
Reasons for Adopting a Pet (*n* = 410)	For support/protection	5	1.2
	Need for friendship	38	9.2
	My children want it	88	21.4
	Because I love animals	279	68.1

This table summarizes participants’ household characteristics, including type of housing, household size, monthly income, pet-related care and feeding expenses, and reasons for pet adoption. All values are reported as frequency (*n*) and percentage (%). Due to rounding, percentages may not add up to exactly 100.

**Table 3:** Information about pets.

Data	Answers	Frequency (*n*)	Percentage (%)
Type of Pet (*n* = 410)	Cat	294	71.7
	Dog	71	17.3
	Both cat and dog	45	11.0
Number of Pets (*n* = 410)	1 Pet	277	67.6
	2 Pets	56	13.6
	3 or more pets	77	18.8
Pet Gender (*n* = 410)	Male	189	46.1
	Female	221	53.9
How Many Years Have You Been a Pet Owner? (*n* = 410)	1–3 years	172	42.0
	4–10 years	168	40.9
	More than 10 years	70	17.1
Pet Neutered? (*n* = 410)	Yes	293	71.5
	No	117	28.5
How Do You Evaluate Your Pet’s Weight? (*n* = 410)	Very weak	1	0.2
	Weak	16	4.0
	Ideal	318	77.6
	Fat	63	15.4
	Overweight	12	3.0

Participants’ responses to the survey question “How do you evaluate your pet’s weight?” reflect their personal assessments. Percentages may not add up to 100 due to rounding.

**Table 4:** Feeding practices and nutritional preferences of pet owners.

Data	Answers	Frequency (*n*)	Percentage (%)
Pet’s Diet (*n* = 410)	Commercial food only	196	47.8
	Only homemade food	16	4.0
	Commercial and homemade food	35	8.5
	Mostly commercial food with some homemade	146	35.6
	Mostly homemade with some commercial food	17	4.1
Preferred Commercial Food Type (*n* = 394) *	Dry foods	375	95.1
	Wet foods	5	1.3
	Semi-watery foods	14	3.6
Preferred Commercial Food Quality (*n* = 394) *	Economical foods	69	17.5
	Premium foods	151	38.3
	Super-premium foods	174	44.2
Where to Purchase Commercial Food (*n* = 394) *	On the internet	177	44.9
	From markets	33	8.4
	From pet shops	88	22.3
	From the veterinary clinic	96	24.4
Reasons for Choosing Commercial Food (*n* = 394) *	Price	19	4.8
	My pet loves	111	28.1
	Content of food	201	51.0
	Brand of food	24	6.1
	Recommendation (friend, veterinarian, etc.)	36	9.1
	Other reasons	3	0.8

* Sixteen participants who preferred only home-cooked food were excluded from this analysis. Daily feeding frequency data corresponding to pet diet categories are illustrated in Figure 2. “Super-Premium” refers to commercial diets formulated with high-quality ingredients and enhanced nutrient profiles; “premium” indicates good quality commercial diets with standard nutrient enhancement; “economical” refers to basic commercial diets with standard nutrient levels.

## Data Availability

The data supporting the findings of this study are available from the corresponding author, S. ÇELİK, upon reasonable request.
